# Causes and Outcomes of Intensive Care Admission Refusals: A Retrospective Audit from a Rural Teaching Hospital in Eastern Cape, South Africa

**DOI:** 10.3390/clinpract13040066

**Published:** 2023-06-25

**Authors:** Ezile Julie Ninise, Busisiwe Mrara, Olanrewaju Oladimeji

**Affiliations:** 1 Anaesthesiology and Critical Care, Faculty of Health Sciences, Walter Sisulu University, Mthatha 5099, South Africa; 2 Department of Public Health, Faculty of Health Sciences, Walter Sisulu University, Mthatha 5099, South Africa

**Keywords:** ICU triage, refusal rates, Nelson Mandela Academic Hospital, a retrospective cross-sectional study

## Abstract

(1) Background: Patients who deserve intensive care unit (ICU) admission may be denied due to a lack of resources, complicating ICU triage decisions for intensive care unit (ICU) clinicians. Among the resources that may be unavailable are trained personnel and monitored beds. In South Africa, the distribution of healthcare resources is reflected in the availability of ICU beds, with more ICU beds available in more affluent areas. Data on ICU refusal rates, reasons for refusal, patient characteristics, and outcomes are scarce in resource-constrained rural settings. Hence, this study sheds light on the ICU refusal rates, reasons for refusal, characteristics, and outcomes of refused patients at NMAH. (2) Methods: This was a three-month retrospective cross-sectional record review of refused and admitted patients from January to March 2022. COVID-19 patients and those younger than 13 years old were excluded. Refusal rates, reasons for refusal, characteristics, and outcomes of refused patients were analysed quantitatively using SPSS VS 20 software. Reasons for refusal were categorised as “too well”, “too sick”, and “suitable for admission but no resources”. (3) Results: A total of 135 patients were discussed for ICU admission at NMAH during the study period; 73 (54.07%) were refused admission, and 62 (45.92%) were admitted. Being considered too sick to benefit from ICU was the most common reason for refusal (53.23%). Too well and no resources contributed 27.42% and 19.35%, respectively. Patients with poor functional status, comorbidities, medical diagnoses, and those referred from the ward or accident and emergency unit rather than the operating room were more likely to be refused ICU admission. Refused patients had a seven-day mortality rate of 47%. (4) Conclusions and recommendations: The study found an unmet need for critical care services at our institution, as well as a need for tools to help clinicians make objective triage decisions for critically ill patients. Therefore, the study suggests a need to improve the quality of services provided outside of the ICU, particularly for patients who were refused ICU admission, to improve their outcomes.

## 1. Introduction

Critical illness is a global health issue of significant magnitude, contributing to the death toll of millions each year. However, this estimate is challenging to pinpoint accurately, as the critical illness is a severe consequence of almost all diseases [1]. At the same time, the availability of critical care beds is severely limited, particularly in low- and middle-income countries (LMICs), due to the costs associated with equipment, technology, and the requisite staffing.

A 2010 study indicated that the proportion of critical care beds in relation to hospital size and population in public hospitals of low- and middle-income countries (LMICs) was less than one-tenth of the ratio seen in High-Income Countries (HICs) [1]. This significant disparity often results in the need for rationing ICU beds. This concept of rationing, as defined by the USA-based Task Force on Values, Ethics, and Rationing in Critical Care (VERICC), involves “the allocation of healthcare resources in situations of limited supply, which unavoidably leads to the denial of certain beneficial treatments to some individuals” [2]. The task force highlighted important ethical dynamics in ICU bed allocation, particularly that decisions based on individual clinical judgement could unmask unethical biases and prejudices. Other authors have highlighted more ethical dilemmas, such as the subjective, situational, and contextual interpretation of concepts such as distributive justice and tools used to objectify the decisions, such as illness severity scores; these could inadvertently worsen inequities in health care [3]. A study on Canadian ICU physicians reported subjectivity in the perception of bed scarcity and how rationing decisions were made [4]; however, there is a dearth of evidence on ICU bed rationing practices among LMIC ICU physicians. ICU capacity limitation is presumed to be the primary reason for admission refusal in LMICs.

South Africa (SA) has a bed-to-patient ratio of 6 per 100,000 population compared to 11.5 beds per 100,000 population in HIC [5]. In 2013, the South African public sector had 25% of the total ICU beds in the country, serving 75% of the population, while the private sector had 75% of the total ICU beds while serving 25%. [6]. In addition, over 75% of the beds were concentrated in the three most affluent provinces: Kwa Zulu Natal, Gauteng, and the Western Cape (3). Thus, ICU physicians in SA public sector hospitals face these difficult decisions regarding ICU bed allocation.

The ICU management model greatly influences decisions about admissions and refusals and can be classified into three models: closed, open, or hybrid. In a closed model, a dedicated ICU specialist, or intensivist, is responsible for all decisions regarding admissions, clinical management, and discharges, which minimizes inconsistencies in patient care. Conversely, in an open model, an intensivist offers optional consultations. At the same time, the primary care physician, such as a surgeon, internal medicine doctor, or family doctor, retains overall responsibility for the patient admitted to the ICU. Scientific evidence indicates that closed units may yield better survival rates than open ones [7,8]. However, in South Africa, only 7% of public units and less than 1% of private units were reported to be “ideal closed units” in 2007 [9]. The impact of these management models on ICU bed rationing practices is unclear, and there is a lack of evidence on the issue. In the closed model, the intensivist has greater responsibility and need for accountability in bed allocation decisions and, as such, is more likely to follow written guidelines.

Despite the diversity in ICU admission practices and the unequal allocation of ICU beds, there’s a conspicuous lack of data regarding refusal rates and the justifications behind these rejections across most South African provinces, including the Eastern Cape. In addition, given the limited number of ICU beds, healthcare providers often employ triage principles to determine which patients stand to gain the most from critical care admission.

Currently, there are no fitting scores for the triage of critically ill patients, prompting the Critical Care Society of Southern Africa (CCSSA) to formulate a consensus guideline on ICU triage and rationing [10]. However, there’s a gap in knowledge about the guideline’s widespread utilization across various units, its impact on refusal rates, and patient outcomes. In addition, research displays a split opinion on the elements that contribute to ICU refusals, with factors such as age, pre-existing conditions, comorbidities, and the patient’s medical status being highlighted as primary influences on the triage decision [11,12]. Yet, Bouneb and his team found no significant discrepancies in age and comorbidities between admitted patients and those who were refused [13].

### Top of Form

This study aimed to examine the rates of refusal for ICU admission, the reasons for refusal, and the clinical outcomes of patients who were refused ICU admission at Nelson Mandela Academic Hospital. Patients who are refused ICU admission may have a higher mortality rate than admitted patients due to differences in care standards between the wards and ICU [13,14]. Additionally, data on the impact of patient care on mortality in patients admitted to general wards is lacking. The study explored the outcomes of refused ICU patients to gain insights into the quality of ward care to aid in motivating the region to increase critical care capacity. The study aimed to describe the causes and characteristics of patients denied ICU care.

## 2. Methods

### 2.1. Ethical Statement

Ethics approval to conduct the study was granted by the Walter Sisulu University Human Research and Ethics Committee on 30 June 2022, approval number 017/2022. Hospital gateway permission to access records was obtained from the Eastern Cape Department of Health on 27 January 2023, approval no EC_202301_010.

As this was a retrospective review of patient records, the ethics committee waived informed consent. Therefore, patient information was kept confidential and de-identified during data collection.

The study has been reported according to the Strengthening the Reporting of Observational Studies in Epidemiology (STROBE) guidelines on reporting observational studies.

### 2.2. Study Design

This was a retrospective observational descriptive cross-sectional study. We reviewed ICU refusal records from 1 January to 31 March 2022. The primary outcome was the refusal rates at the NMAH adult ICU. Secondary outcomes were factors influencing refusal rates at NMAH adult ICU, the characteristics of patients refused ICU admission and the outcomes of refused patients within seven days.

### 2.3. Study Setting

Nelson Mandela Academic Hospital (NMAH) is a provincial government-funded hospital in the South African town of Mthatha. It is part of the Mthatha Hospital Complex. NMAH is the only tertiary hospital in the Eastern Cape’s north-eastern region. In comparison to other tertiary hospitals, NMAH is located in the province’s rural areas and serves a catchment area of approximately three million people that stretches along the North-Eastern part of the Eastern Cape Province

Most of the population comprises rural, black patients from poor socio-economic backgrounds. Nelson Mandela Academic Hospital is the only referral hospital for this population for surgical procedures and ICU care. The hospital has 736 beds, including an Orthopaedic unit nine kilometres away.

This study focused on the adult ICU which has an 8-bed capacity. The ICU is one of the sub-speciality units in the hospital, which has a bed capacity of 6–8 ICU beds and six high-care beds. The ICU works with nurse: patient ratios 1:1 for ventilated ICU patients and 1:2 for high-care patients. It is a closed ICU run by a critical care specialist and a team of Anaesthesiologists; referred patients are reviewed by the critical care team, with the final decision to admit made by the critical care consultant on-call. The hospital has separate specialized adult and paediatric units at the time of the study, including a Paediatric ICU, a COVID-19 ICU/High Care, Trauma high care, and Obstetrics high care.

### 2.4. Population

The hospital serves a catchment area of approximately three million people. This single-centre study focused on adult patients admitted to the ICU over January, February, and March 2022.

Inclusion Criteria

- Patients above 12 years of age. According to the agreed scope between the paediatric department and the adult specialities, the adult ICU at NMAH cares for patients 13 years old and above.
- Patients requiring ICU admission as per referring doctor and refused as per ICU team.
- Patients refused ICU admission at NMAH from 1 January to 31 March 2022. The area of focus was the reasons for refusal, patients’ characteristics, and outcomes of refused patients. We also calculated the refusal rate during the study period.

Exclusion criteria

- Records missing the most relevant parameters.
- COVID-19 patients (COVID-19 patients are admitted in a different ward and section of the hospital to the non-COVID-19 ICU ward, with other staff and resources).

### 2.5. Sampling Methods

Convenience sampling of data on all patients discussed for possible ICU admission was done. Both admitted and refused patient data were extracted from an ICU consult book. Patients who met the criteria for the study during the specified period were included. A fraction of refused patients taken from the total number of referred patients were used to calculate refusal rates.

### 2.6. Data Sources

Pre-existing data was extracted from the ICU consult book used by the ICU team.

Data in the book includes dates, referral departments, patient’s demographics (name, file number, diagnosis, age, and gender), reasons for referral and reasons for refusal, etc., as adapted from the ICU proforma form. For data extraction for the study, the data was de-identified.

### 2.7. Sample Size Calculation

A sequential retrospective review of all refused patients, as recorded in the ICU logbook, was performed. In addition, patients’ records who met the study’s criteria during the specified period were included—objective one required including all patients in the sample frame to estimate the refusal rate with near accuracy. However, for all of the objectives, we needed a sample size estimate [15].

Patients refused ICU admission at NMAH from 1 January 2022 to 31 March 2022 comprised the study population. Using an extrapolated proportion of patients who were refused ICU admission at NMAH of 60% with a 95% confidence interval and 10% margin of error, approximately 101 [92/(1 − 0.1)] records of patients who were refused ICU admission were planned for review. An additional 10% was set aside to account for incomplete records.

### 2.8. Data Collection Instrument, Entry and Analysis

Relevant patient data were de-identified and assigned unique numbers. After that, data was recorded in the ICU consult book for each patient discussed for admission. This data was extracted from the ICU consult book to achieve the stated objectives.

All data collected were entered into an Excel program for arrangement, analysed with the help of a statistician, and exported to a software program for further statistical analysis. Categorical data were summarised as proportions or percentages. Continuous data were compared using a *t*-test, while categorical data were compared using the Chi-Square or Fischer exact. Statistical significance was defined as a *p*-value of <0.05.

## 3. Results

### 3.1. Primary Outcome

**Figure 1:**
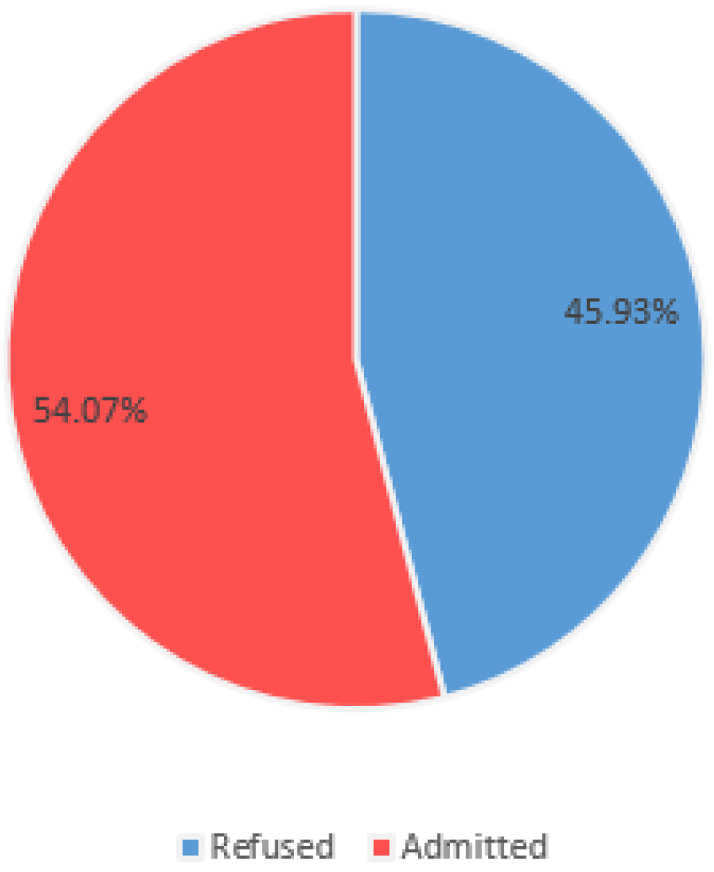
Decision at triage: Refusal rate at NMAH adult ICU.

The majority of patients that were referred to NMAH adult ICU were admitted. The refusal rate is shown in Figure 1.

### 3.2. Secondary Outcomes

Demographics and clinical characteristics of refused and admitted patients in ICU.

The mean age was 36.78 ± 17.16 years in the admitted group and 41.27 ± 19.68 years in the refused group. There was no statistically significant difference. Most admitted patients were females (37), and males comprised the majority in the refused group (34).

Table 1 presents the socio-demographic characteristics. Most of the ICU consults in the cohort were in the 30–39 age group, followed by the 20–29 age group, with a comparable number in the admitted and refused groups. More referrals were accepted in March (26), with more refusals in January (29).

Among the admitted patients, the commonest referring unit was the operating theatre (43). In comparison, the Accident & Emergency unit was the commonest referral site for the refused group of patients (43). In addition, more patients were admitted from the general surgery department (39) compared to other departments, whereas most refused patients were from the internal medicine department (37). Due to the younger age of the cohort, most patients in the study had a normal functional status.

Table 2 provides information about the clinical conditions of patients who were admitted and those who were refused from the ICU. For those admitted, sepsis was the most common primary disease (28), while patients with other pathologies comprised most of the refused group (31). In addition, it was observed that patients with surgical diagnoses were more likely to be admitted, whereas those with medical conditions were more likely to be refused. (36). These findings were statistically significant.

Infectious diseases such as human immunodeficiency virus (HIV) and tuberculosis were more likely to be found in admitted patients compared to those rejected (21). Cardiovascular diseases such as hypertension and congestive cardiac failure (15), together with endocrine pathologies such as diabetes mellitus (8), were more common among the refused group. These findings were also statistically significant.

### 3.3. Reasons for Refusal

**Figure 2:**
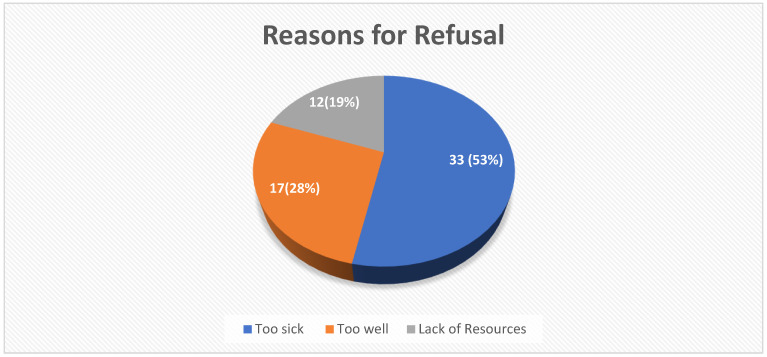
Reasons for Refusal.

Regarding the reasons for refusal, 62 patients were assessed. Figure 2 shows the distribution of refused patients according to reasons for refusal.

**Figure 3:**
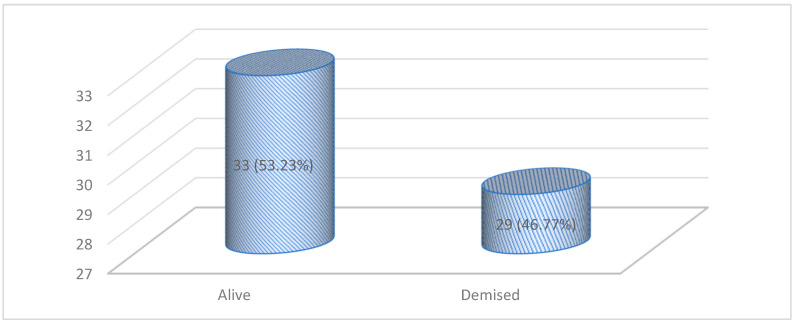
Seven-day outcomes of patients refused admission.

The seven-day outcomes of patients who were refused admission are shown in Figure 3.

Most patients who were refused due to being too sick, died (28). Next, most patients refused due to being too well, survived (16). Finally, all patients who were refused admission due to lack of resources, were alive at seven days. See Table 3.

## 4. Discussion

One hundred and thirty-five patients were referred to our ICU during the study period, and 45.9% were refused ICU care. Patients deemed too sick at triage were more likely to be rejected. A poor functional status, comorbidities, a medical diagnosis, and referral from the ward or accident and emergency unit were likely to lead to a refusal decision. Finally, 47% of the patients who were refused admission died within seven days of the decision. The majority of deaths occurred in the “too sick” category of patients, with only one patient dying in the “too well” category and no death occurring in the “no resources” category.

### 4.1. ICU Refusal

This study found a 45.9% refusal rate for ICU admission, which is consistent with international refusal rates in both HIC and LMIC. In HIC, lower refusal rates have been documented; Robert et al. documented a refusal rate of 14.48% in France, while Orsini et al. documented a refusal rate of 57% in the United States of America [16,17]. In LMICs such as South Africa, Gordon et al. and Gopalan with De Vasconcellos found refusal rates of 28% and 38.7%, respectively [18,19].

In Tunisia, another LMIC, Bouneb et al. found 79.5% refusal rates [13]. In these international studies, the average age of rejected patients is around 65 years old, whereas rejected patients in our study were younger (20–39 years) [16,17,20]. This demonstrates that, while ICU bed rationing is a global issue, it adversely affects younger patients who require ICU admission in poorer regions.

### 4.2. Reasons for Refusal

The reasons for refusal were presented as multiple-choice items, such as “too sick to benefit from ICU care”, “too well to benefit from incremental care”, and “suitable for admission but lack of resources (no beds or nurses)”. Unfortunately, to the authors’ knowledge, no tools in the literature could be adapted, introducing subjectivity into how patients are classified at triage by the ICU team.

### 4.3. Too Sick to Benefit from ICU Admission

“Too Sick/ill” means patients with a very low life expectancy due to acute and/or chronic illnesses present at triage, as determined by the ICU physician. Patients deemed too sick to benefit from ICU care comprised the majority of ICU refusals, accounting for 53.23%. This is similar to other studies [14,18,19]. Among the factors influencing this decision were the severity of the illness, comorbidities, poor functional status, and medical diagnosis.

### 4.4. Too Well to Need ICU Care

In our study, this category was the second most common reason for refusal of ICU admission, with a rate of 27.42%, indicating that inappropriate referral is a concern in our setting and high-income countries. Education, triage scoring systems, and guidelines could thus assist local clinicians in making the best decision when referring patients to ICU care. 

Clinicians may also refer these patients to the intensive care unit (ICU) if they believe it will provide better monitoring and clinical management than the general ward. Staff issues such as nurse-to-patient ratios, skill, adequate training, and knowledge were identified by Mselle and Msengi as challenges in managing critically ill patients in the ward [21]. It was also reported that the general wards lacked specialised equipment to monitor and manage these patients, such as ventilators, infusion pumps, and basic oxygen delivery systems.

When dealing with patients believed to require close monitoring and advanced care, clinicians’ distress and discomfort may be exacerbated. On the other hand, strategies for increasing surveillance and care for these patients in general wards can be implemented. In general wards, early warning scores may aid in early detection, accelerating the implementation of life-saving interventions, and motivating ICU triage. In a Portuguese study, Correia et al. found that early warning scores increased the likelihood of receiving early medical attention, predicted patient deterioration, and the need for a high-care unit [22].

### 4.5. No Resources

In contrast to the findings of LMIC studies by Bouneb et al. and Merhabene et al., our study found that a lack of resources (no beds, a shortage of nurses, etc.) was not the primary reason for ICU refusal [13,20]. Instead, this reason had the least influence on whether a patient was refused or admitted to the ICU, accounting for 19.35% of all refused patients. This is most likely due to our institution’s efforts to increase ICU capacity, such as contracting extra nurses, obtaining extra equipment from other units, and optimising remote environments with equipment to accommodate these patients.

### 4.6. Characteristics of Patients Who Were Refused ICU Admission

Several studies have found that old age is associated with ICU refusal [12,16,18,23]. Our findings are consistent with those of Bouneb et al., who found no relationship between age and the triage decision in their study [13].

It should be noted that our ICU referral cohort was generally young, so there was no age-based decision-making conflict. Our institution rejected more patients in the first two months of the year, with more admissions in March, which could be attributed to a high rate of critically ill trauma patients being referred to the ICU during the holiday season.

In their study, Gopalan and De Vasconcellos found no correlation between seasonal variation in patient illness and triage decisions [18]. However, the return of elective schedules, the opening of outpatient clinics at the beginning of the year, and the rush to clear surgical wards may have influenced the number of patients referred to the ICU during the first three months of the year.

Regarding referral sites and departments, we tended to refuse more patients referred directly from wards and the accident and emergency unit, but we had a lower threshold for admitting those referred from the operating room. The most likely reason is that patients from the operating room typically have interventions that improve their overall prognosis. In contrast, patients from the ward and accident and emergency unit usually have a medical diagnosis and, in some cases, end-stage disease, which ICU clinicians view as poor prognostic factors. As a result, we admit more surgical patients than medical patients.

Our findings agree with those of Gopalan and De Vasconcellos, who found that being referred from a medical discipline was a significant factor in ICU admission refusal [18]. However, Orsini et al. found that surgical patients had a higher likelihood of ICU refusal than medical patients, but their explanation for this finding was unclear [17]. Patients with poor functional status were more likely to be denied ICU admission. Our findings support the findings of Sprung et al. and Escudero-Acha et al., who suggested that poor functional status is associated with ICU refusal [23,24].

Our study found that patients with unknown premorbid functional status were likely to be refused ICU care. The most likely reason for refusal of unknown functional status was clinicians’ proclivity to associate unknown functional status with a high likelihood of poor prognosis. In their study of patients with unknown premorbid functional status, Gopalan and De Vasconcellos had similar findings [18]. Unfortunately, there is currently no literature to assist in making triage decisions on patients with unknown functional status, as this requires knowledge of daily activity before ICU triage.

Comorbid illnesses and a primary medical diagnosis were more likely to result in ICU refusal. This is consistent with previous studies that found that medical patients with comorbid illnesses were more likely to be refused ICU care [12,16,18,23]. Similar to our findings, Gopalan and De Vasconcellos found comorbidities such as HIV, malignancy, and cardiac failure as significant factors in ICU admission refusal in their study [18]. Surprisingly, Bouneb et al. found no important link between comorbidities and ICU refusal [13].

Our study found that HIV-positive status was less likely to result in ICU refusal. Only eight of the 24 patients who were discussed for admission were refused ICU admission; the rest were admitted. This contrasts with the findings of Gopalan and De Vasconcellos, who revealed that HIV status was a significant factor in ICU refusal [18]. A possible explanation for this finding is that the patients admitted to our unit were not classified as having acquired immunodeficiency syndrome (AIDS) as per World Health Organization (WHO) definitions.

This practice is consistent with the critical care society of South Africa’s consensus guideline on ICU triage and admission, which states that patients should not be discriminated against solely because of their HIV status, especially if the reason for admission is unrelated to the underlying retroviral disease [10].

Regarding the primary disease, trauma and septic patients were more likely to be admitted due to the assumption that their illness was reversible. Conversely, patients in other categories in our study were more likely to be rejected, including cancer and other chronic end-stage illnesses deemed of poor prognosis by ICU clinicians.

### 4.7. 7-Day Outcomes of Refused Patients

46.77 percent of patients who were refused admission died within seven days of being denied admission. This is due to less intensive monitoring and interventions outside the ICU. Hurri et al., in their study, found that only 54.4% of refused patients were alive by 30 days [14]. In our study, patients classified as “too sick to benefit from ICU admission” had the highest mortality rate (84%) within seven days of refusal.

These findings are comparable to those made by Bouneb et al., who found a mortality rate of 80% for those refused ICU admission due to being “too sick to benefit from ICU” [13]. They found that the mortality rate in those categorised as “too well to benefit from ICU” was 5.71%, similar to the 5.88% in our study [13]. They also found that those who were refused due to a lack of beds died at a rate of 26.56%, contrary to our findings, as no death was reported following the refusal of admission due to a “lack of resources” [13].

The reasons for a zero-mortality rate for patients denied admission due to a lack of resources may be due to sourcing skilled nurses, equipment, and improved monitoring to optimise ward care for these patients. At the same time, they wait for a bed in the ICU for later admission.

In our study, one patient died in the ward in the “too well” category. As the triage assessment may be flawed and have some subjectivity, this can result in disastrous consequences. Additionally, when a patient is determined “too well to benefit from ICU”, ward personnel may believe that no additional measures beyond standard ward care are required to improve the care of such a patient.

### 4.8. Limitations

Due to the retrospective observational nature of the study, determining the actual reasons for refusal was not always easy. This limitation also applies to important details regarding the patient’s diagnosis, including the source of sepsis.

The sampling method used was convenience sampling with no randomization, introducing selection bias.

This is a single-centre study with a relatively small sample size, limiting the generalizability of the study findings.

The categorisation and outcomes of admitted patients were not reported to compare and highlight the association of ICU care refusal with increased mortality.

## 5. Conclusions

This study has revealed that our institution’s capacity to provide critical care services is suboptimal, with almost a fifth of the refusals stemming from a lack of resources in the form of staffed ICU beds. The study also affirmed the subjectivity and inherent bias of triage decisions. Therefore, developing triage decisions based on scoring systems adaptable to low-resource areas could also be a powerful tool to guide clinicians dealing with this issue daily.

The high mortality rate of refused patients suggests inadequate ward care and skills to manage these patients. Given these subjective triage decisions, patients could be wrongly classified, and some could do well in better-resourced health systems. Therefore, step-down wards with monitoring, basic ventilation, and hemodynamic support, as well as trained personnel, would be of benefit. This is important to augment the insufficient ICU facilities. Improving ICU capacity, including increasing bed numbers and staff skills for the hospital and the region, is an urgent need, as is developing surge capacity and preparing for increased referrals during pandemics and disasters.

There is a need to enhance care systems outside the ICU to reduce the demand for ICU beds and improve the ward care of patients who cannot be admitted to the ICU. Ward care could be improved with focused training, monitoring of vitals using early warning scoring systems, and rapid response teams. These interventions could aid in the early recognition, triaging, resuscitation, and stabilization of ICU patients. Lastly, a large multi-centre study would be beneficial in elucidating trends in rationing practices in underserved areas.

## Figures and Tables

**Table 1:** Characteristics of patients refused and admitted to ICU.

Variables	Status		(*p*-Value)
	Admitted (*n* = 73)	Refused (*n* = 62)	
	*n* (54%)	*n* (45.9%)	
Age			
≤19	12 (16.44)	4 (6.45)	
20–29	15 (20.55)	14 (22.58)	
30–39	21 (28.77)	18 (29.03)	4.43 (0.489) ^χ2^
40–49	12 (16.44)	9 (14.52)	
50–59	6 (8.22)	7 (11.29)	
≥60	7 (9.59)	10 (16.13)	
Mean (SD)	36.78 ± 17.16	41.27 ± 19.68	1.42 (0.159) ^µ^
Gender			
Male	36 (49.32)	34 (54.84)	0.41 (0.522)
Female	37 (50.68)	28 (45.16)	
Weekday			
Monday	17 (23.29)	14 (22.58)	
Tuesday	12 (16.44)	16 (25.81)	
Wednesday	8 (10.96)	12 (19.35)	0.183 ^γ^
Thursday	13 (17.81)	7 (11.29)	
Friday	7 (9.59)	8 (12.90)	
Saturday	6 (8.22)	1 (1.61)	
Sunday	10 (13.43)	4 (6.45)	
Month			
January	22 (30.14)	29 (46.77)	
February	25 (34.25)	27 (43.55)	12.72 (0.002) *
March	26 (35.62)	6 (9.68)	
Site of referral			
Accident & Emergency	15 (20.55)	43 (69.35)	
Ward	15 (20.55)	18 (29.03)	0.001 *^,γ^
Operating Theatre	43 (58.73)	1 (2.27)	
Department of Referral			
General Surgery	39 (53.42)	19 (30.65)	
Internal Medicine	15 (20.55)	37 (59.68)	
Neuro-Surgery	5 (6.85)	3 (4.84)	0.001 *^,γ^
Obstetrics & Gynaecology	10 (13.70)	1 (1.61)	
Orthopedics	2 (2.74)	2 (3.23)	
Urology	2 (2.74)	0 (0.0)	
Functional Status			
Normal	51 (69.86)	30 (48.39)	
Poor	3 (4.11)	9 (14.52)	0.020 *^,γ^
Unknown	19 (26.03)	23 (37.10)	

* Statistically significant (*p* < 0.05); ^χ2^ = Chi-Square; ^γ^ = Fisher’s Exact *p*: ^µ^ = Student *t*-test.

**Table 2:** The clinical condition of patients refused and admitted to ICU.

Variables	Status		(*p*-Value)
	Admitted (*n* = 275)	Refused (*n* = 136)	
	*n* (%)	*n* (%)	
Primary Disease			
Trauma	25 (34.25)	15 (24.19)	
Sepsis	28 (38.36)	16 (25.81)	7.30 (0.026) *^,χ2^
Other	20 (27.40)	31 (50.0)	
Primary Diagnosis			
Medical	20 (27.40)	36 (58.06)	12.99 (0.001) *^,χ2^
Surgical	53 (72.60)	26 (41.94)	
Co-morbidities (*n*_1_ = 52, *n*_2_ = 68)			
Infectious Disease	21 (40.38)	10 (14.71)	
Cardiovascular Disease	15 (28.85)	26 (38.24)	
Endocrine	8 (15.38)	13 (19.12)	
Respiratory disease	1 (1.92)	7 (10.29)	0.027 *^,γ^
Kidney disease	4 (7.69)	6 (8.82)	
Cancer	0 (0.0)	3 (4.41)	
Other	3 (5.77)	3 (4.41)	

* Statistically significant (*p* < 0.05); ^χ2^ = Chi-Square; ^γ^ = Fisher’s Exact *p.*

**Table 3:** Seven-day outcomes of patients refused admission by reasons for refusal.

Reasons for Refusal	Outcome		Total	χ2 (*p*-Value)
	Alive	Demised		
Too sick	5 (15.15)	28 (84.85)	33 (100.0)	
Too well	16 (94.12)	1 (5.88)	17 (100.0)	0.001 *^,γ^
Lack of Resources	12 (100.0)	0 (0.0)	12 (100.0)	

* Statistically significant (*p* < 0.05); χ2 = Chi-Square; ^γ^ = Fisher’s Exact *p.*

## Data Availability

Clean datasets used for this study are not publicly available. However, the authors could share this upon reasonable request and with the permission of the Nelson Mandela Academic Hospital and Walter Sisulu University.
